# The Gene *FvTST1* From Strawberry Modulates Endogenous Sugars Enhancing Plant Growth and Fruit Ripening

**DOI:** 10.3389/fpls.2021.774582

**Published:** 2022-01-11

**Authors:** Arif Rashid, Haixiang Ruan, Yunsheng Wang

**Affiliations:** ^1^College of Life Science, Anhui Agricultural University, Hefei, China; ^2^State Key Laboratory of Tea Plant Biology and Utilization, Anhui Agricultural University, Hefei, China

**Keywords:** *FvTST1*, sugar, fruit ripening, *PIFs*, auxin signaling pathway, growth regulation

## Abstract

Sugar is an important carbon source and contributes significantly to the improvement of plant growth and fruit flavor quality. Sugar transport through the tonoplast is important for intracellular homeostasis and metabolic balance in plant cells. There are four tonoplast sugar transporters (*FvTST1-4)* in strawberry genome. The qRT-PCR results indicated that *FvTST1* has a differential expression pattern in different tissues and developmental stages, and exhibited highest expression level in mature fruits. The yeast complementation assay showed that *FvTST1* can mediate the uptake of different sugars, such as fructose, glucose, sucrose, and mannose. Subcellular localization analyses revealed that *FvTST1* was mainly targeted to the tonoplast. Transient expression of *FvTST1* in strawberry fruits enhanced both fruit ripening and sugar accumulation. Furthermore, *FvTST1*-transformed tomato plants exhibited higher sucrose and auxin content, enhanced seed germination and vegetative growth, higher photosynthetic rate, early flowering, and bore fruit; fructose and glucose levels were higher in transgenic fruits than those in the control. Transcriptomic analysis indicated that the auxin signaling pathway was highly enriched pathway in up-regulated Gene-ontology terms. In transgenic plants, genes encoding transcription factors, such as phytochrome-interacting factors *PIF1, -3*, and *-4*, as well as their potential target genes, were also induced. Collectively, the results show that *FvTST1* enhances plant growth and fruit ripening by modulating endogenous sugars, and highlight the biological significance of this gene for future breeding purposes.

## Introduction

Sugars play pivotal roles in plant growth and development. They are a carbon source and signal molecules regulating multiple gene networks (Ruan, 2014). Sugars are synthesized in photosynthetic leaves (source) and eventually transported to nonphotosynthetic tissues (sink) such as those in roots and fruits, where they are used as a carbon source for plant growth and fruit development (Tognetti et al., 2013; Julius et al., 2017). Sucrose (Suc), monosaccharide fructose (Fru), and glucose (Glu) are important for improving fruit quality, because they act as primary nutrients and sweetening compounds (Patrick et al., 2013). Although the sugar content of a fruit is primarily dependent on leaf (source) input, the key in regulating sugar accumulation lies within the fruit (Ruan and Patrick, 1995).

Vacuoles account for nearly 90% of entire cellular volume in mature fruits and store more than 70% of total sugar (Shiratake and Martinoia, 2007). Plant cell activity to accumulate sugar is regulated by sugar flow in the cytoplasm and the function of sugar transporters in the tonoplast (Ruan, 2014; Ren et al., 2018; Zhu et al., 2021). The tonoplast consists of a group of transporters, including SUTs/SUCs (sucrose transporters) (Schneider et al., 2012), SWEETs (Klemens et al., 2013; Eom et al., 2015), VGTs (vacuolar glucose transporters) (Aluri and Büttner, 2007), and TSTs (Wormit et al., 2006), and it encodes proteins consistent with sugar influx and efflux into plant vacuoles.

Recently, many reports have demonstrated that the TST family of sugar transporters is important for sugar accumulation in plants. For instance, *PpTST1* expression tendency is associated with accumulation of sugar in fruits, and its TRV-mediated suppression inhibited sucrose and hexose content in peach fruits (Peng et al., 2020). In pear, six *PbTMT*s were reported, of which *PbTMT4* was highly expressed in the fruit. Moreover, the heterologous expression of *PbTMT4* in tomato significantly enhanced Glu and Fru content in tomato fruits (Cheng R. et al., 2018), suggesting that *PbTMT4* likely transports hexoses. *CmTST2* expression was higher in sugar-rich varieties and was lower in low-sugar varieties in melon, and its overexpression dramatically increased sugar content in strawberry and cucumber fruits (Cheng J. et al., 2018). *MdTST1* expression was higher during apple fruit development, indicating its functional correlation with sugar influx to vacuoles during the ripening of the fruit (Wei et al., 2014). In grape fruits, *VvTST1* and *VvTST2* were significantly expressed TST isoforms closely associated with hexose accumulation (Afoufa-Bastien et al., 2010; Çakir and Giachino, 2012). The overexpression of *Arabidopsis TST*s (*AtTST1* and -2, formerly named as *TMT1* and *-2*) facilitates sugar influx into plant vacuoles (Wormit et al., 2006; Schulz et al., 2011). Sugar signaling plays a crucial role in plant growth (Mishra et al., 2021) and enhances the ripening of fruits (Jia et al., 2013; Durán-Soria et al., 2020). For example, exogenous sucrose treatment promoted fruit coloration, ripening, and postharvest process of strawberry and tomato fruits (Jia et al., 2013; Li et al., 2016). Recent research documented that water melon TST *(ClTST2)* overexpression is associated with sugar accumulation, and that it is controlled by a sugar-induced transcription factor (*SUSIWM1*) (Ren et al., 2018). The sugar-inducible transcription factor *SUSIWM1* induces *ClTST2* transcript level by binding to the SURE element in the promoter region, thereby enhancing sugar influx into the vacuole. Intriguingly, in potato, the SURE element in a gene promoter was regulated by sucrose, which suggests that during fruit ripening, storage of sugars in the vacuole is mediated by *TST*s and regulated by sucrose (Grierson et al., 1994). Sucrose crosstalk with plant hormones has also been documented, such as the transcription factors *PIF*, which has recently been identified to be crucially implicated in sucrose signaling (Lilley et al., 2012), directly induces the transcript level of expansins (*EXP*s) and *XYLOGLUCAN ENDOTRANSGLUCOSYLASE/HYDROLASE PROTEIN*s (*XTH*s). Many plant hormones, including auxin, brassinosteroid (BR), ethylene (ET), and gibberellic acid (GA) (Kohnen et al., 2016; Ivakov et al., 2017), are involved in this process.

The study reports the expression of *FvTSTs* in the strawberry fruit, and vegetative as well as reproductive tissues, and determined the highest expression level of *FvTST1* in mature fruits. The transport function of *FvTST1* was analyzed in *hxt-null* yeast mutant. Moreover, *FvTST1* was overexpressed in strawberry fruits and tomato plants to elucidate its physiological role. An RNA-sequences analysis was performed to examine significant transcriptional changes between transgenic and non-transgenic tomato plants.

## Materials and Methods

### Plant Material

Strawberry seeds (*Fragaria vesca* L.) were grown in a mixture of peat, vermiculite, and perlite (volume ratio of 3:2:1) by regular fertilization. Fruits were harvested in different developmental stages and were grouped as previously reported (Jia et al., 2013); small green (about 6 days after flowering (DAF), big green (13 DAF), degreening (18 DAF), white (22 DAF), initiated redness (26 DAF), partially red (29 DAF), and fully red (33DAF); and were stored at –80°C for qPCR analyses. The vegetative tissues including root, stem, leaf bud, first leaf, second leaf, and mature leaf, as well as reproductive tissues such as flower buds, initiated opening, partial opening, and fully opened flowers, were also collected and frozen on-site in liquid nitrogen, then stored at −80°C until use.

### RNA Extraction, Synthesis of cDNA, and *FvTST1* Cloning

Total RNA was extracted from specific tissues using RNAprep Pure Kit (Takara, Beijing, China) (for polysaccharide- and polyphenolic-rich plants), following the user manual from the manufacturer. Nanodrop 2000 Spectrophotometer (Thermo Fisher Scientific, Wilmington, DE, United States) and 1% agarose gel electrophoresis were used to determine RNA quality and concentration. The extracted RNA was digested with *DNase* I, and PrimeScript™ RT Master Mix (Takara, Beijing, China) was used to synthesize cDNA.

The open reading frame of the cDNA was amplified with gene-specific primers (Supplementary Table 2) based on FvTST1 (Accession XM_004300064) available at NCBI. The whole cDNA of *FvTST1* was then amplified with mix super fidelity polymerase (Vazyme Biotech Co., Ltd.), under thermal cycling conditions of 95°C denaturation for 5 min, followed by 30 cycles of 95°C for 30 s, 60°C for 30 s, 72°C for 2 min, and a final extension of 72°C for 10 min. A single band of expected size 2217 bp was purified from gel and sub cloned into pEASY vector (TransGen Biotech, Beijing, China).

### Quantitative Real-Time PCR

In this study, qPCR was conducted for *FvTSTs* in different tissues of strawberry, and for validation of some genes between transgenic and non-transgenic lines (control). We designed the primers with the Primer Premier 5.0 software (Premier Biosoft, Palo Alto, CA, United States) (Supplementary Table 2). The *18S* ribosomal RNA (*18S* rRNA) (accession number KF668233.1) was used for transcript level normalization. PrimeScriptTM RT Kit (Perfect Real Time) (Takara, Beijing, China) was used for the preparation of the qPCR reaction, and the CFX96 platform (Bio RAD, Shanghai, China)^1^ was used to perform the reaction. The 2^–ΔΔCT^ method was used for the calculation of the transcript levels of the genes.

### Yeast Mutant Complement Assay

The Sugar transport function was elucidated in yeast mutant strain (EBY.VW4000) (Wieczorke et al., 1999) using lithium acetate transformation with pYES2.0 vector containing the *FvTST1* gene or with empty vector (Gietz et al., 1992). *FvTST1* was amplified using a pair of primers with restriction sites *Sac* I and *Xba* I at their 5′ ends (Supplementary Table 3). To get pYE2.0-*FvTST1*, the resulting DNA fragment was cleaved with *Sac* I and *Xba* I, and was connected into pYES2.0. The pYES2.0-*FvTST1-*transformed yeast strain (EBY.VW4000) and transformants were cultured and selected as reported before (Li et al., 2017). For complementation assay of sugar, yeast cells were inoculated in a liquid medium with 2% maltose as a carbon source, and were incubated overnight at 30°C to an optical density (OD_600_) of 1. The yeast cells were then diluted serially with (10-, 100-, and 1,000-fold), and a 4-μl dilution was cultured in the medium containing yeast nitrogen base without uracil with 2% maltose, fructose, glucose, sucrose, and mannose as carbon sources. Wild-type yeast was used as positive control, while an empty vector and EBY.VW.4000 were used as negative controls. All the plates were placed at 30°C for 2 days and were documented.

### Subcellular Localization

The *FvTST1* gene fused with GFP was cloned into the binary vector *pBI121*. The CaMV35s-FvTST1-GFP fusion construct, tonoplast marker CaMV35s- γTIP-mCherry (CD3-975), and plasma membrane marker CaMV35s-PIP2A-mCherry (CD3-1007) (Nelson et al., 2007) were verified by sequencing, and then transformed into *Agrobacterium*. The *Agrobacterium* was inoculated in an LB medium and incubated overnight at 28°C in a shaker at 200 rpm. The bacterial culture was collected and then suspended in a buffer containing MES; 10 mM (pH 5.6), MgCl_2_ 10 mM, and acetosyringone 200 mM to an OD_600_ of 1. The mixture was kept at room temperature for 1–3 h and was infiltrated into the lower surface of *Nicotiana benthamiana* leaves with a 1-ml syringe. After 3 days, the infiltrated leaves were put on water glass slides for microscopic examination. Fluorescence was detected in leaf cells using a TCS-SP8 laser-scanning confocal microscope (Leica, Mannheim, Germany). The fluorescence detection wavelengths of GFP were 488 and 500–530 nm, and the fluorescence detection wavelengths of mCherry were 514 and 560–620 nm.

### Construction of Plant Overexpression Vector

The CDS of *FvTST1* was sub-cloned into the binary vector *pBI121* employing *Xba* I and *Sac* I. The promoter of the TOMATO PROLINE RICH PROTEIN (TPRP-F1) gene (X61395.1) (Salts et al., 1991) was then placed 5′ upstream of the *FvTST1* CDS in *pBI121-FvTST1* using a pair of one-step recombinant cloning primers (Supplementary Table 2) employing *Hind* III and *Xba* I to get the plant expression construct *pBI121-P_*TPRP–F1*_::FvTST1.*

### Transient Expression of *FvTST1* in Strawberry Fruit

*FvTST1* and its counterpart empty vector *pBI121* were infiltrated into strawberry fruits according to a previous report (Jia et al., 2013). An LB liquid medium with antibiotics was used for the inoculation of *Agrobacterium* and incubated overnight at 28°C to an optical density (OD_600_) of 1. The culture was collected and then suspended in the buffer (10 mM MgCl_2_, 10 mMMes, pH5.6, and 200 mM acetosyringone) to an OD_600_ = 0.8, and placed at room temperature for 2 h before infiltration into the fruit. Fruits of strawberry attached to the plant in degreening stage (18 DAF) were injected using a sterile 1-ml hypodermic syringe.

### Generation of Transgenic Tomato Plants

To generate the *FvTST1* transgenic tomato, *Agrobacterium-*mediated leaf disk transformation of tomato [*Solanum lycopersicum* cv. Ailsa Craig+ (AC+)] was performed as reported previously (Sun et al., 2006). Cotyledons of 2-week-old seedlings were pre-cultured in an MS medium supplemented with 0.1% Gamborg’s vitamins, 1 μg/ml 6-BA, and 40 ng/ml of IAA for 3 days. The cotyledons were then immersed for 15 min in an *Agrobacterium* culture (OD600 = 0.1–0.2) with the liquid MS medium. The cotyledons were dried and then co-cultured for 2 days in an MS medium containing 0.1% Gamborg’s vitamins, 0.1 μg/ml Kinetin, 0.2 μg/ml 2, 4-D, and 1.5 μg/ml acetosyringone under dark conditions. After co-cultivation, the cotyledons were transformed into a regeneration and selection MS medium (0.1% Gamborg’s vitamin, 10 μg/ml 6-BA, 0.4 μg/ml IAA, 0.5 mg/ml carbenicillin, and 50 mg/ml kanamycin). After every 3 weeks, the medium was refreshed until shooting. Two-to-three-centimeter newly developed shoots were then transformed into a rooting MS medium (0.1% Gamborg’s vitamins, 4 μg/ml IAA, 0.5 mg/ml carbenicillin, and 50 mg/ml kanamycin) until true roots developed. The presence of *FvTST1* in positive regenerants from kanamycin selection was verified by PCR and sequencing.

### Transcriptome Sequencing, Differentially Expressed Genes, Gene Ontology, and KEGG Analysis

The T1 seeds from T0 transgenic tomato plants from each independent transgenic line were geminated in a 1/2 MS medium containing kanamycin, and the non-transgenic seeds were grown in a 1/2 MS medium without kanamycin and transferred to a growth chamber, which was set at 25°C/22°C with relative humidity of 65%, a photoperiod of 16 h and a photon flux of about 200 μmoles⋅m^–2^⋅s^–1^. Seven-day-old (4 DAG) transgenic and control seedlings were collected, frozen in liquid nitrogen, and stored at –80°C until use. The total RNA from independent transgenic and control lines was extracted. RNA-seq was performed with a commercial RNA-seq agent (OE Biotech, Shanghai, China), and with Trimmomatic; the raw data (raw reads) were analyzed (Bolger et al., 2014). To obtain the clean reads, poly-n and low-quality reads were removed. Clean reads were mapped to reference genomes of *Solanum lycopersicum* cultivar “Heinz 1706,” available at the Sol Genome network ITAG v2, using HISAT2 (Kim et al., 2015). Cufflinks were used for normalization of exonic reads (Trapnell et al., 2012), which were reported as FPKM, and the number of gene reads was counted using htseq-count (Anders and Huber, 2012; Anders et al., 2015). DESeq R package functions, estimate size factors, and nbinomTest were used to find DEGs (Anders and Huber, 2012). A *p* value < 0.05 and a fold change > 2 or < 0.5 have been set as the significant differential expression thresholds for the selection of induced or suppressed genes. The DEGs were analyzed for GO and KEGG pathway enrichment using the R package (Kanehisa et al., 2008), which was based on hypergeometric distribution.

### Measurement of Soluble Sugars

The contents of Fru, Glu, and Suc, in the strawberry fruit, tomato seedlings, and fruits were determined using an LC-20A high-performance liquid chromatography system (Shimadzu Company, Kyoto, Japan) with a 5-μm XB-NH2 column (4.6 mm × 250 mm; Shanghai Welch Materials, China) and a RID20A detector. To develop a standard curve, authentic standards of different sugars were purchased from Sigma (St Louis, MO, United States). Soluble sugars, Fru, Glu, and Suc were extracted according to previous reports (Castonguay et al., 1995). Plant tissues weighing 0.5 g were ground with liquid nitrogen, and soluble sugars were extracted using 10 ml of distilled water at 100°C for 1 h. The samples were centrifuged at 13,000 g for 10 min, and the supernatant was transferred to a new 10-ml tube and brought to 10 ml with distilled water. A 1-ml sample was filtered before HPLC analysis through a 0.22-μm filter membrane. The mobile phase contained acetonitrile/water with a volume-to-volume ratio of 75:25 v/v, and flow rate was adjusted to 1 ml/min with an injection volume of 10 μl.

### Measurement of Auxin

The IAA authentic internal standard (BCCC6104) was purchased from Sigma-Aldrich (St. Louis, MO, United States. IAA was extracted following previous reports (Pan et al., 2008). Seedlings weighing 0.3 g were ground to fine powder with liquid nitrogen. Extraction buffer (isopropanol–HCl, 2: 0.002 v/v) with 2 ml was added to the powder and incubated for 30 min at 4°C and centrifuged at 13,000 g for 5 min, and repeated twice. After centrifugation, the lower organic phase was transferred to a 10-mL tube and evaporated in a constant stream of nitrogen. The samples were kept in the dark before being resuspended in a solution of 150 μl methanol and 0.1% formic acid. The samples were filtered with a 0.45-μm microfilter, and the reversed phase column (C18 ZORBAX 300SB, 3 μm; 4.6 mm × 9 mm × 150 mm; Agilent, Sta. Clara, CA, United States) was used for sample injection. The mobile phase contained methanol as solvent A, water with 0.1% formic acid was used as solvent B, and column temperature was adjusted to 30 °C. The product was purified using a solvent gradient, which began with volume adjustment to 20% methanol for 2 min, and was linearly increased to 80% methanol for 14 min, after equilibration for 5 min, and then reverted to the volume of 20% methanol for 0.1 min. A hybrid triple quadruple mass spectrometer (SCIEX 6500 QTrap; Applied Biosystems, Foster City, CA, United States) was employed for quantification analysis. System voltage was set to 4.5 kV. Source temperature was set to 500°C. Nebulizer and curtain drying gas pressure was adjusted to 75.15 and 65 psi, respectively.

### Measurement of Photosynthesis

The net photosynthesis rates of cotyledons of (4 DAG) seedlings were measured with CIRAS-3 (PP Systems, Amesbury, MA, United States). The analyzers act as absorption meters to measure infrared absorption. The optical bench is temperature-controlled and pressure-compensated to provide accurate CO_2_ and H_2_O measurement. Net photosynthesis (A) was determined from the difference between the CO_2_ concentration entering (Cin) and exiting (Cout) the cuvette. IRGA CO_2_ reading was corrected for water vapor, temperature, and atmospheric pressure. Since humidity dilutes the air leaving the cuvette (Cout), it was compensated using the following equation: net photosynthesis (A) = (Cin × W) – [Cout × (W+E)], where (A) is net photosynthesis, (W) is mass flow of air per unit leaf area into the cuvette, and (E) is transpiration rate.

### Statistical Analysis

The experiment was conducted using a completely random method. For statistical analysis, the SPSS software version 19.00 (SPSS, Chicago, IL, United States) was used. The mean values of transgenic lines and wild type lines were compared by independent *t-*test or one-way ANOVA test. The data was expressed as the mean ± standard deviation of three replicates.

## Results

### Expression Analysis of *FvTST*s in Different Tissues

**FIGURE 1:**
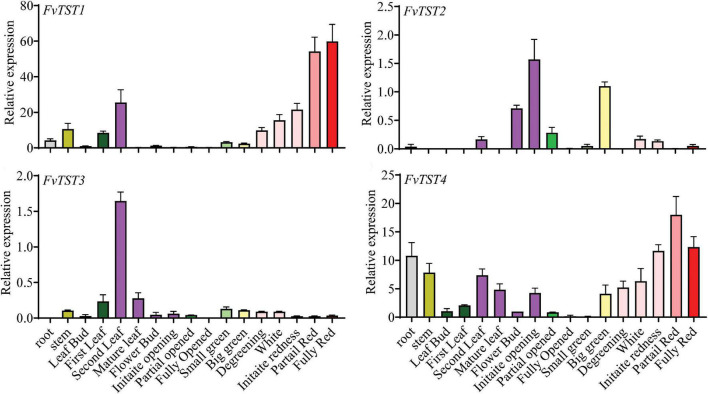
Transcript profile of *FvTSTs* in different organs of strawberry in different developmental stages by quantitative real-time polymerase chain reaction (qRT-PCR). The data represent mean ± standard deviation of three biological replicates. To normalize the relative expression level, *18S rRNA* was used as an internal control.

Previously, four putative TST genes, *FvTST1-4*, were identified in the strawberry genome (Jiu et al., 2018; Liu et al., 2020). The phylogenetic tree revealed that *FvTST*s share significant homology with *ArabidopsisAtTST1, -2, -3* (Supplementary Figure 1). To understand the possible role of *FvTST*s, the expression profile of these genes was analyzed in different tissues by qRT-PCR analysis (Figure 1). Expression analyses of *FvTSTs* indicated that they were differentially expressed in all the examined tissues in different developmental stages. Among them, *FvTST1* was strongly expressed in the second leaf; however, it was weakly expressed or unnoticeable in other tissues, including young fruits, but its level was significantly increased in fully red fruits. Moreover, *FvTST2* and *FvTST3* had almost the same weak expression tendencies during plant growth and fruit development. On the contrary, *FvTST4* expression was detected in the root, stem, and leaf, and was weakly expressed in flower buds, partially open flowers, fully open flower, and small green fruits, while higher expression level was detected in partially red fruits, and then decreased in fully red fruits. Overall, the results highlight that *FvTST1* is a highly expressed TST gene during strawberry fruit development, and suggest that it might contribute to sugar accumulation in fruits.

### Heterologous Expression of *FvTST1* in Yeast System

**FIGURE 2:**
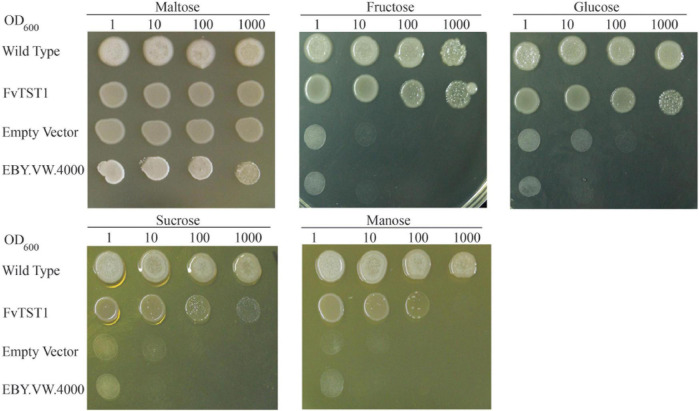
Sugar transport function analysis of *FvTST1* in yeast mutant strain EBY.VW.4000. Yeast cells were diluted serially (10-, 100-, and 1,000-fold) and were spotted in a minimal solid medium for 2 days at 30°C, with 2% maltose, fructose, glucose, sucrose, and mannose as sources of carbon. The wild-type yeast was used as a positive control, and EBY.VW.4000 and empty vector were used as negative controls.

The expression profile of genes is used as a useful tool for the selection of candidate genes; however, further evaluation is necessary. In a widely used heterologous system, the transport function of many sugar transporters has been characterized (Lemoine, 2000). The *hxt-null* strain EBY.VW4000 (Wieczorke et al., 1999; Conde et al., 2007) has been widely used for studying plant sugar transporters. The knockout of hexose transporters in this strain prevents it from uptaking hexoses, thus allowing the assessment of the gene introduced. *FvTST1* was expressed in EBY.VW4000 *hxt-null* yeast mutant cells. Selection of the transformant was carried out in the medium containing maltose without uracil as a carbon source, and was confirmed by PCR (Supplementary Figure 2). The pYES2.0–FvTST1 transformant in the yeast strain was then plated in a medium containing 2% maltose, fructose, glucose, sucrose, and mannose as key carbon sources. The positive control was wild-type yeast, and the negative control was an empty vector and EBY.VW.4000. The serial dilution (10-, 100-, and 1,000-fold) of yeast strain demonstrated that the EBY.VW4000 transformant with pYES2.0–FvTST1 and wild-type yeast can grow in all the media including the sucrose-containing medium, and that the empty vector and EBY.VW.4000 can only grow in the presence of maltose (Figure 2). This result confirms that *FvTST1* is a functional tonoplast sugar transporter.

### Subcellular Localization of *FvTST1* Proteins

**FIGURE 3:**
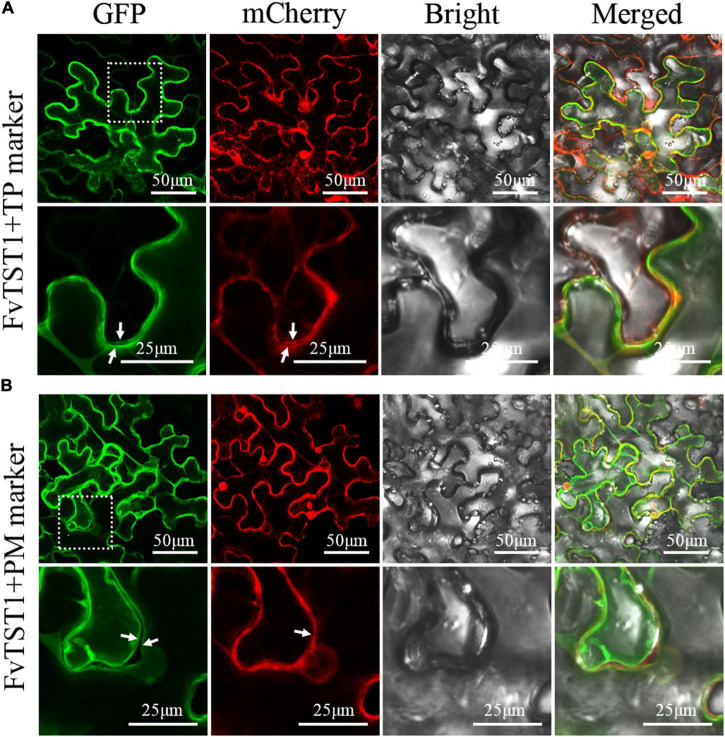
Subcellular localization of FvTST1-GFP fusion protein in *Nicotiana benthamiana*. **(A)** Co-localization of FvTST1-GFP with Gamma TIP-mCherry (CD3-975 tonoplast, TP, marker) in *N. benthamiana* leaves. **(B)** Co-localization of *FvTST1-*GFP and PIP2A-mCherry (CD3-1007; plasma membrane, PM, marker) in *N. benthamiana* leaves. The dashed box indicates the enlarged portion and highlights localization differences between TP and PM.

For subcellular localization of *FvTST1*, we established a construct encoding *FvTST1-GFP* driven by a 35S promoter, and was co-expressed wit hCaMV35s-γTIP-mCherry (tonoplast marker, CD3-975) and CaMV35s-*PIP2A*-mCherry (plasma membrane marker, CD3-1007) (Nelson et al., 2007), in *Nicotiana benthamiana* leaves. Confocal microscopy images show that the GFP signal from *FvTST1* overlapped with the mCherry signal of the tonoplast marker, and a merged yellow signal was observed in whole cells, whereas in zoom-in cells, two GFP lines and two mCherry lines from the tonoplast marker were observed and indicated by white arrows in zoom-in cells (Figure 3A). We further analyzed the co-localization of *FvTST1* with the plasma membrane marker. Two linear GFP signals (white arrows), a single mCherry signal, and a weak merged yellow signal were observed in the zoom-in cells of the plasma membrane marker (Figure 3B). These data show that *FvTST1* is mainly located in the tonoplast.

### Overexpression of *FvTST1* in Strawberry Enhances Sugar Accumulation and Fruit Ripening

**FIGURE 4:**
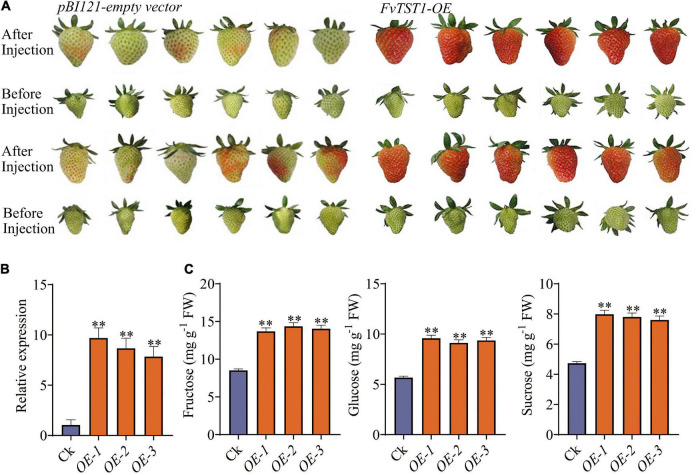
Transient expression of *FvTST1* in strawberry fruit. **(A)** Phenotypes of *FvTST1-*overexpressed strawberry fruit. **(B)** qRT PCR analyses of *FvTST1*-overexpressed fruit and control. **(C)** Sugar content (fructose, glucose, and sucrose) in *FvTST1*-overexpressed fruits. The values represent mean ± standard deviation of three biological replicates. The SPSS software (v19.0) was used for one-way analysis of variance (ANOVA) (^**^p < 0.01).

To elucidate the possible role of *FvTST1* during fruit development, the construct with *FvTST1* and *pBI121* empty vectors (control) were injected in the degreening stage (18 DAF) of strawberry fruits, while the fruits were still attached to the plants. Seven days after injection, we observed that the fruits injected with *FvTST1* turned red, and that the fruits with the *pBI121* empty vector did not turn red (Figure 4A). We analyzed the expression level of *FvTST1* in these two groups and found that *FvTST1* exhibited higher expression in the *FvTST1*-injected fruits than in the control (Figure 4B). The sugar concentration in these two groups was assessed, and the content of Fru, Glu, and Suc was significantly induced in the *FvTST1*-overexpressed fruits than in the control (Figure 4C). These findings demonstrated that *FvTST1* overexpression in the strawberry fruits enhanced fruit ripening and sugar accumulation.

### Transgenic Tomato Expressing *FvTST1* Exhibited Enhanced Growth and Sugar Accumulation

**FIGURE 5:**
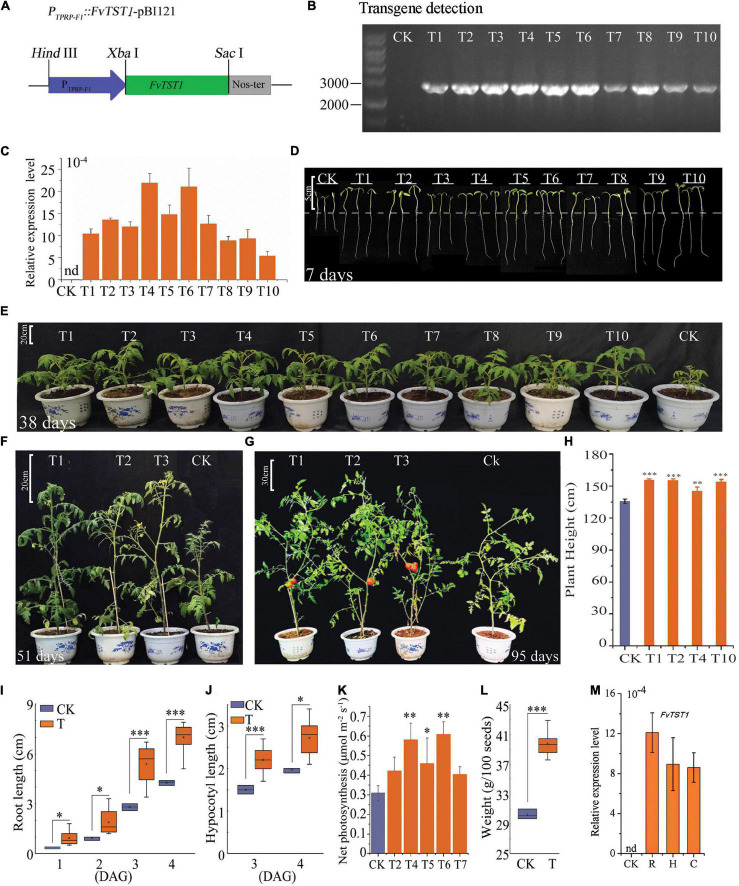
Phenotypic alteration of *FvTST1* transgenic tomato (*Solanum lycopersicum* cv. Ailsa Craig+ (AC+). **(A)** *P_*TPRP–F1*_::FvTST1-pBI121* plants expression construct. **(B)** PCR detection of *FvTST1* in transgenic plants. **(C)** Transcript levels of *FvTST1* in transgenic and control plants. **(D)** Phenotypic differences among 7-day-old (4 DAG) transgenic plants (T) compared to control (CK) (Scale bar = 5 cm). **(E)** Enhanced growth of 38-day-old transgenic plant compared to control (Scale bar = 20 cm). **(F)** Phenotypes of 51-day-old transgenic plants and control (Scale bar = 20 cm). **(G)** Phenotypes of 95-day-old transgenic and control plants (Scale bar = 30 cm). **(H)** Plant height differences between transgenic plant and control. **(I)** Root growth of the 7-day-old (4 DAG) transgenic plants compared to control. **(J)** Hypocotyl growth of 7-day-old (4 DAG) transgenic plants compared to control. **(K)** Net Photosynthesis rate **(L)** Higher seed weight of transgenic tomato compared to control plants. **(M)** Transcript level analysis of *FvTST1* in root (R), hypocotyl (H) and cotyledon (C). (CK refers to control and “T” stands for transgenic, and number represents independent transgenic line). The data were shown as mean ± standard deviation of three biological replicates. The SPSS software (v19.0) was used for one-way ANOVA (*p < 0.05; ^**^p < 0.01; ^***^p < 0.001).

To further elucidate the role of *FvTST1* in plant growth and fruit development, the *FvTST1* overexpression construct (Figure 5A) was overexpressed in the tomato plant. Transgene presence in the transgenic plants was analyzed by PCR (Figure 5B), and a high level of transgene transcript was detected in all the independent transgenic lines, but not detected in the non-transgenic (control) line (Figure 5C). In particular, the transgenic plant shows enhanced vegetative growth (Figures 5D,E), flowers, and bears fruits (Figures 5F,G) significantly earlier than control. Transgenic plant height was significantly higher than that of the control (Figure 5H). Moreover, to understand the phenotypic alteration between transgenic plants and control, the seedlings were characterized further. The root and hypocotyl length of “transgenic seedlings” was significantly longer (*p* < 0.05) than that of control (Figures 5I,J). Moreover, higher net photosynthetic rates were observed in some of the transgenic lines (T4, T5, and T6) than in the control (Figure 5K). Transgenic seeds were heavier than the control (Figure 5L), and they germinated 2 days earlier than the control. *FvTST1* transcript level was significantly increased in the root, hypocotyl, and cotyledons of the transgenic plants, but it was not detected in the control plants (Figure 5M).

**FIGURE 6:**
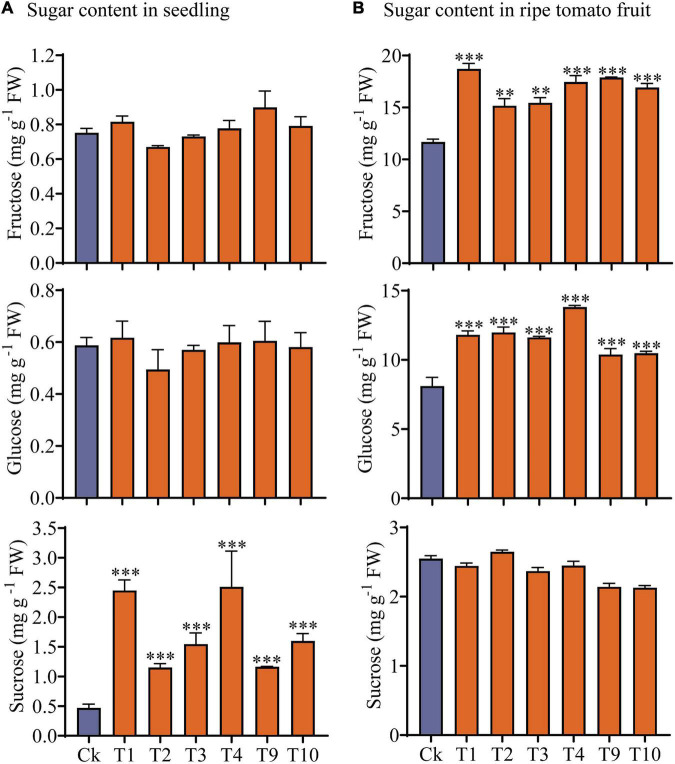
Analysis of sugars content in transgenic and control plants. **(A)** Sugar content in 7-day-old (4 DAGs) transgenic plants and control plants. **(B)** Sugar content in mature fruit of transgenic and non-transgenic plants. Values are mean ± standard deviation of three replicates. Statistical analysis was performed with the SPSS software (v19.0); one-way *ANOVA* followed by Dennett’s multiple comparisons was performed (^**^p < 0.01; ^***^p < 0.001).

Soluble sugars (Fru, Glu, and Suc) were measured in seedlings and mature fruits of the transgenic and control plants (Figure 6). Our results showed that Suc content was significantly higher in the transgenic seedlings than in the controls, but that there were no obvious changes in Fru and Glu content (Figure 6A). However, when the transgenic plant mature fruits were compared to the controls, the Fru and Glu contents were significantly increased, but no changes in Suc content were observed (Figure 6B).

### Transcriptomic Alteration in Transgenic Tomato Seedlings

RNA-seq analysis was performed to reveal the transcriptional changes in the transgenic plants. Clean reads ranged from 46.35 to 49.29 M, total mapped reads were 97%, and Q30 of the raw bases was 94% (Supplementary Table 1), suggesting that the transcriptome data were reliable. A total of 25,934 genes were expressed in the 7-day old (4 DAG) tomato seedlings. Among the DEGs, using fold change > 2 and a Q-value < 0.05, a total of 1,142 genes were up-regulated and 746 genes were down-regulated (Supplementary Figure 3A). According to GO analysis, the down-regulated genes had 514 enriched (*q* < 0.05) GO terms, whereas the up-regulated genes had 427 enriched (*q* < 0.05) GO terms.

Gene ontology (GO) was further studied, and the top 10 GO terms of biological process (BP), cellular component (CC), and molecular function (MF) for up-regulated and down-regulated genes were analyzed (Supplementary Figures 3B,C). In the up-regulated biological process, “the auxin signaling pathway,” “auxin polar transport,” and “regulation of growth” were highly enriched terms (Supplementary Figure 3B). Apoplast, membrane system, and cell wall were enhanced, which was consistent with enhanced seedling growth (Supplementary Figure 3B). PSI was the most affected pathway in BP, CC, and MF (Supplementary Figure 3C) in the down-regulated genes. Low-affinity nitrate transmembrane transport was found to be down-regulated (Supplementary Figure 3B), suggesting alteration of nitrate/carbon balance. The genes selected during this study are available at https://solgenomics.net/ while the name and accession numbers are provided (Supplementary Table S3).

### Transcriptional Alterations of *Phytochrome-Interacting Factor*s, Their Putative Target Genes and Auxin Signaling Pathway Enhanced Hypocotyl Elongation, Root Growth

**FIGURE 7:**
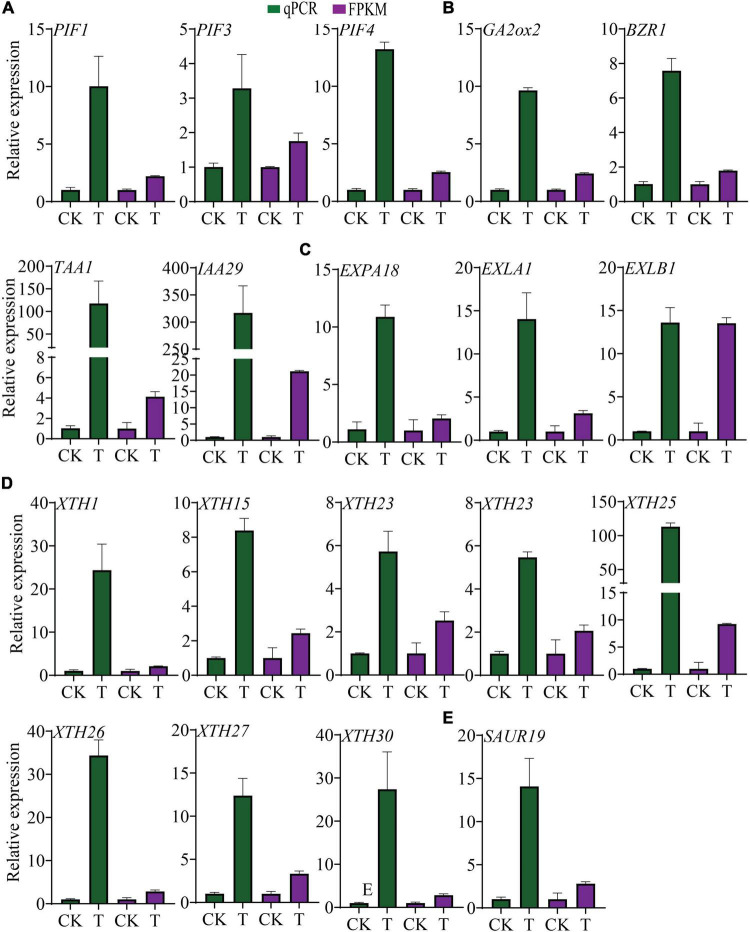
Transcriptional alterations of *PIFs*, their putative target genes and hypocotyl and root growth-related DEGs. **(A)** Expression level of *PIF1, -3, -4* in transgenic plants and control. **(B)** Expression of *PIF* putative direct target genes *GA2ox2*, *BZR1* and *TAA1*, and *IAA29.* **(C)** Transcript levels of expansins. **(D)** Transcript level of *XTH*s. **(E)** Transcript level of *SAUR19*. The data were shown as mean ± standard deviation of three biological replicates, and *18srRNA* was used as internal control for the normalization of expression level.

*Phytochrome-interacting factor*s (*PIFs*) are a group of negative regulators of photomorphogenesis (Toledo-Ortiz et al., 2010; Kim k. et al., 2016) and are induced by sucrose (Lilley et al., 2012). In this study *PIF1*, *-3*, and *-4* were significantly up-regulated, and were confirmed by qPCR analysis (Figure 7A). PHYTOCHROME B (PHYB), which is suppressed by *PIFs* (Jang et al., 2010), was found down-regulated with a fold change of 0.62. Many genes that were directly induced by *PIF*s were significantly enhanced in this study, such as *BZR1*, *GA2ox2*, *TAA1*, and *IAA29* (Toledo-Ortiz et al., 2010), and their expression was also confirmed by qPCR (Figure 7B). *PSY*, another target of *PIF1* (Toledo-Ortiz et al., 2010), was also found to be down-regulated. *PIF*s have the ability to bind to G-box, E-box, and ACE *cis*-elements (Zhang et al., 2013; Kim J. et al., 2016), and the existence of G-box, E-box, and ACE was checked across the 2kb segment upstream of the translation start site (ATG) for *GA2ox2*, *BZR1*, and *TAA1*. The results showed that 5, 8, and 18 *cis*-elements existed in the promoters of *TAA1*, *BZR1*, and *GA2ox2*, respectively (Supplementary Figure 4A). Moreover, sequences of the 2kb segment upstream of the ATG of all 1,888 DEGs from the tomato genome were examined for *cis*-element analysis. The results revealed that most of the promoters of the examined DEGs contained E-boxes and ACE. The promoters of the genes containing G-boxes accounted for about 1/4 to 1/3 of the total number of the affected genes (Supplementary Figure 4B).

In addition, *PIF*s and auxin both regulate *EXP*s and *XTH*s, which are important for hypocotyl elongation and root growth. In this study, 6 *EXP*s and 10 *XTH*s were up-regulated, except *XTH8*, *XTH32*, and *EXPA1* (Figures 7C,D). Besides, the transcription of the gene *SAUR19* was up-regulated (Figure 7E), which has been reported previously for hypocotyl growth stimulation indirectly regulated by *PIF*s (Leivar and Monte, 2014).

**FIGURE 8:**
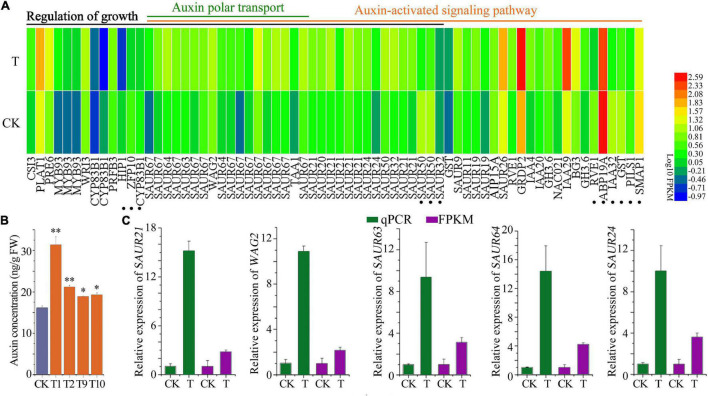
Auxin signaling pathway was affected in the *PTPRPF1::FvTST1* lines **(A)** DEGs related to “Regulation of growth,” “Auxin-activated signaling pathway,” and “Auxin polar transport.” **(B)** Auxin concentration in transgenic seedlings and control. **(C)** qPCR verification of several genes involved in auxin signaling pathway. The dot represents the twelve genes that are responsible for growth enhancement, but not the members of auxin signaling pathway. Values are means ± standard deviation of three biological replicates. The SPSS software (v19.0) was used for one-way ANOVA (*p < 0.05; ^**^p < 0.01).

Interestingly, a total of 54 DEGs were obtained in the “auxin-activated signaling pathway,” with 45 up-regulated and 9 down-regulated, including *SAUR9/11/20/21/24/26/32/50/63/64*, and *WAG2*. Regarding “Auxin polar transport,” which is vital for root growth (Sabatini et al., 1999; Grieneisen et al., 2007; Gutierrez et al., 2012), linked DEGs were all up-regulated (Figure 8A). Consistently, a significantly higher auxin concentration was found in the 7-day old (4DAG) transgenic seedlings compared to the control (Figure 8B). It was worth noting that 33 DEGs out of 45 “growth regulation” genes were members of the auxin signaling pathway, indicating the pivotal role of auxin in regulating transgenic plant growth. Expression alterations of *SAUR19/21/24/63/64*, *WAG2*, were further confirmed by qPCR analysis (Figure 8C). These findings strongly suggest that *PIF1, -3, -4* and/or auxin signaling pathways are involved in enhancement of root growth and hypocotyl elongation.

## Discussion

High sugars accumulate in many fleshy fruits, such as apple, grape, tomato, and strawberry. In fruit cells, the vacuole is the major compartment for sugar storage in fruit cells, accounting for more than 90% of total cell volume (Shiratake and Martinoia, 2007; Hedrich et al., 2015). The tonoplast comprises many sugar transporters including SWEETs (Eom et al., 2015), SUCs/SUTs (sucrose transporters) (Schneider et al., 2012), VGTs (Aluri and Büttner, 2007), and TSTs (Wormit et al., 2006). Tonoplast sugar transporters (Wormit et al., 2006) are the most important regulators that contribute to sugar influx into the vacuole. Several *TST*s have been reported in diverse plant species that influenced plant growth and fruit quality, including *Arabidopsis* (Wormit et al., 2006), grape (Afoufa-Bastien et al., 2010; Çakir and Giachino, 2012), apple (Wei et al., 2014), melon (Cheng J. et al., 2018), watermelon (Ren et al., 2018), and pear (Cheng R. et al., 2018). However the understanding of the TST function in different plant species is underestimated. The findings of this study provide insights into the role *FvTST1* in sugar accumulation in fruit and plant growth enhancement.

### The Role of *FvTST1* in Sugar Accumulation in Strawberry Fruit

There are 4 *TSTs* in the strawberry genome. In particular, *FvTST1* shares significant homology with previously identified sugar transporters such as *AtTST1* (Wormit et al., 2006), *VvTST1* (Afoufa-Bastien et al., 2010), and *MdTST1* (Wei et al., 2014). The expression levels of these genes have been analyzed in different tissues (Figure 1). *FvTST1* was highly expressed during strawberry fruit development, and its expression pattern was consistent with fruit development and sugar accumulation, warranting speculations that *FvTST1* may play a role in strawberry fruit sugar accumulation. These assertions coincide with previous findings (Wei et al., 2014; Cheng J. et al., 2018).

In addition, to identify the function of *FvTST1* as a sugar transporter, we overexpressed *FvTST1* in hexose-deficient yeast (EBY.VW4000) (Wieczorke et al., 1999). The yeast expressing gene *FvTST1* can grow in the medium containing, fructose, glucose, sucrose, and mannose, but the hexose-deficient yeast (EBY.VW4000) and empty vector did not grow (Figure 2), which revealed that *FvTST1* is a functional sugar transporter. Our results are consistent with previous findings that were observed for the plasma membrane transporter and tonoplast transporter (Vignault et al., 2005; Cheng R. et al., 2018). In our study, *FvTST1* transported sucrose, and our findings are concomitant with that of *ArabidopsisAtTMT1,-2-*transfected cells (Wormit et al., 2006; Schulz et al., 2011), which are located into the tonoplast, and have hexose and sucrose import activity. Furthermore, we have analyzed the subcellular localization of *FvTST1* in *N. benthamiana* leaves (Figure 3), indicating that FvTST1-GFP fluorescence is mainly observed in the tonoplast, which coincides with previous studies such as on *AtTST1* (Wormit et al., 2006) and *CmTST2* (Cheng J. et al., 2018).

In addition, to elucidate the physiological function and substrate specificity of *FvTST1 in vivo*, we transiently overexpressed *FvTST1* in the strawberry fruit. The fruits overexpressing *FvTST1* gene matured faster, and exhibited higher sugar content than the control (Figure 4), confirming the function of *FvTST1* as a sugar transporter, and promoting strawberry fruit maturation. In our study, the gene *FvTST1*-overexpressed fruits showed early maturity, and this alteration could be related to the involvement of sucrose signaling, because in previous studies it was documented that the content of sucrose in the cytosol has signaling functions regulating strawberry fruit maturation (Jia et al., 2013). The overexpression of *FaSUT1*, a sucrose transporter in the plasma membrane of strawberry, enhanced sucrose content and fruit ripening, but the down regulation of this gene reduced sucrose content and ripening of fruit (Jia et al., 2013).

### Heterologous Expression of Strawberry *FvTST1* in Tomato Modulates Endogenous Sugar and Promotes Seedling Growth *via* Auxin Signaling Pathway

To assess the role of *FvTST1* in plant and fruit development, we observed the phenotypic differences between *FvTST1*-overexpressing tomato plants and control. The overexpression of *FvTST1* exhibited visible phenotypes, such as fast vegetative growth, early flowering, and enhanced fruit ripening (Figure 5), as those observed for tomato plants overexpressing *PbTMT4* (Cheng R. et al., 2018). Particularly, in *FvTST1-*overexpressed transgenic seedlings and fruits, the content of sucrose and hexose were significantly elevated (Figure 6), respectively. It has been reported that the overexpression of *MdTST1* and *MdTST2* can increase soluble sugar content in apple callus, and that *MdTST1* and *MdTST2* RNAi suppression significantly reduced soluble sugars (Zhu et al., 2021). Previous studies have shown that the overexpression of *AtTST1, -2*-altered sugar in subcellular compartments enhanced seed biomass and early plant development (Wingenter et al., 2010; Schulz et al., 2011). However, in *Arabidopsis*, the overexpression of *AtTST1* has no effect on the abundance of soluble sugars in transgenic seedlings (Wingenter et al., 2010). Nevertheless, in this study, relative to the non-transgenic tomato seedlings, the transgenic tomato seedlings exhibited a higher level of sucrose but not hexose (Fru or Glu). These findings suggest that *TST* genes are conserved in many plant species, and that they are likely to have functional differences in different plants species and e1ven distinct tissues.

To understand further the underlying mechanism of *FvTST1* during plant growth and development, RNA sequencing analysis was performed. Auxin activation pathway genes were found to be significantly enriched, indicating that the auxin signaling pathway is essential for seedling development. Previous research studies have indicated that Suc can induce seedling growth through auxin-mediated signaling pathways (Mishra et al., 2009; Lilley et al., 2012). The sucrose-induced auxin signaling pathway involves *IAA29*, *YUC8* and the transcription factor *phytochrome-interacting factor*s (*PIF*s) (Lilley et al., 2012). In our study, *FvTST1* overexpression enhanced sucrose content in transgenic seedlings and found that *PIF1*, –*3*, and –*4*, were significantly up regulated, which coincides with previous findings (Lilley et al., 2012). In addition to many up-regulated genes, *BZR1* and *GA2ox2*, *TAA1*, and *IAA29* regulated by *PIF*s are crucial for the BR, GA, and auxin signaling pathways (Leivar and Monte, 2014), and sucrose-induced seedling growth (Lilley et al., 2012).

## Conclusion

The present data demonstrated that *FvTST1* was highly expressed predominantly in fully red fruits, and highlighted its functional association with fruit ripening. Furthermore, *FvTST1* transports fructose, glucose, sucrose, and mannose in the *hxt-null* mutant, and its overexpression in strawberry fruit, tomato plant, and localization experiments support its function as a tonoplast sugar transporter, as well as its importance for sugar accumulation in fruits. Moreover, RNA-seq analyses unravel the mechanism of fast growth and development, indicating that the up-regulation of auxin signaling pathway-linked genes was the most significant transcriptional change. The transcript level of *PIF1*, *-3*, and *-4* was induced in the transgenic seedlings, which highlighted the role of the sucrose signaling pathway. Collectively, our results suggest that *FvTST1* is a consideration target for manipulating early fruit bearing plants and fruits with sweet flavor.

## Data Availability Statement

The original contributions presented in the study are included in the article/Supplementary Material, further inquiries can be directed to the corresponding author.

## Author Contributions

AR generated the transgenic plants and performed the main experiments, analyzed the transcriptomic data, and prepared the manuscript draft. HR critically reviewed the manuscript. YW conceived the project and finalized the manuscript. All authors contributed to the article and approved the submitted version.

## Conflict of Interest

The authors declare that the research was conducted in the absence of any commercial or financial relationships that could be construed as a potential conflict of interest.

## Publisher’s Note

All claims expressed in this article are solely those of the authors and do not necessarily represent those of their affiliated organizations, or those of the publisher, the editors and the reviewers. Any product that may be evaluated in this article, or claim that may be made by its manufacturer, is not guaranteed or endorsed by the publisher.

## Footnotes

1
www.bio-rad.com
