# Supplementary material for: The Gene FvTST1 From Strawberry Modulates Endogenous Sugars Enhancing Plant Growth and Fruit Ripening
Source: Front Plant Sci. 2022 Jan 11;12:774582. doi: 10.3389/fpls.2021.774582 (PMC8786802; doi:10.3389/fpls.2021.774582)
Supplement: Supplementary file 1 [file Data_Sheet_1.docx]

Supplementary Material


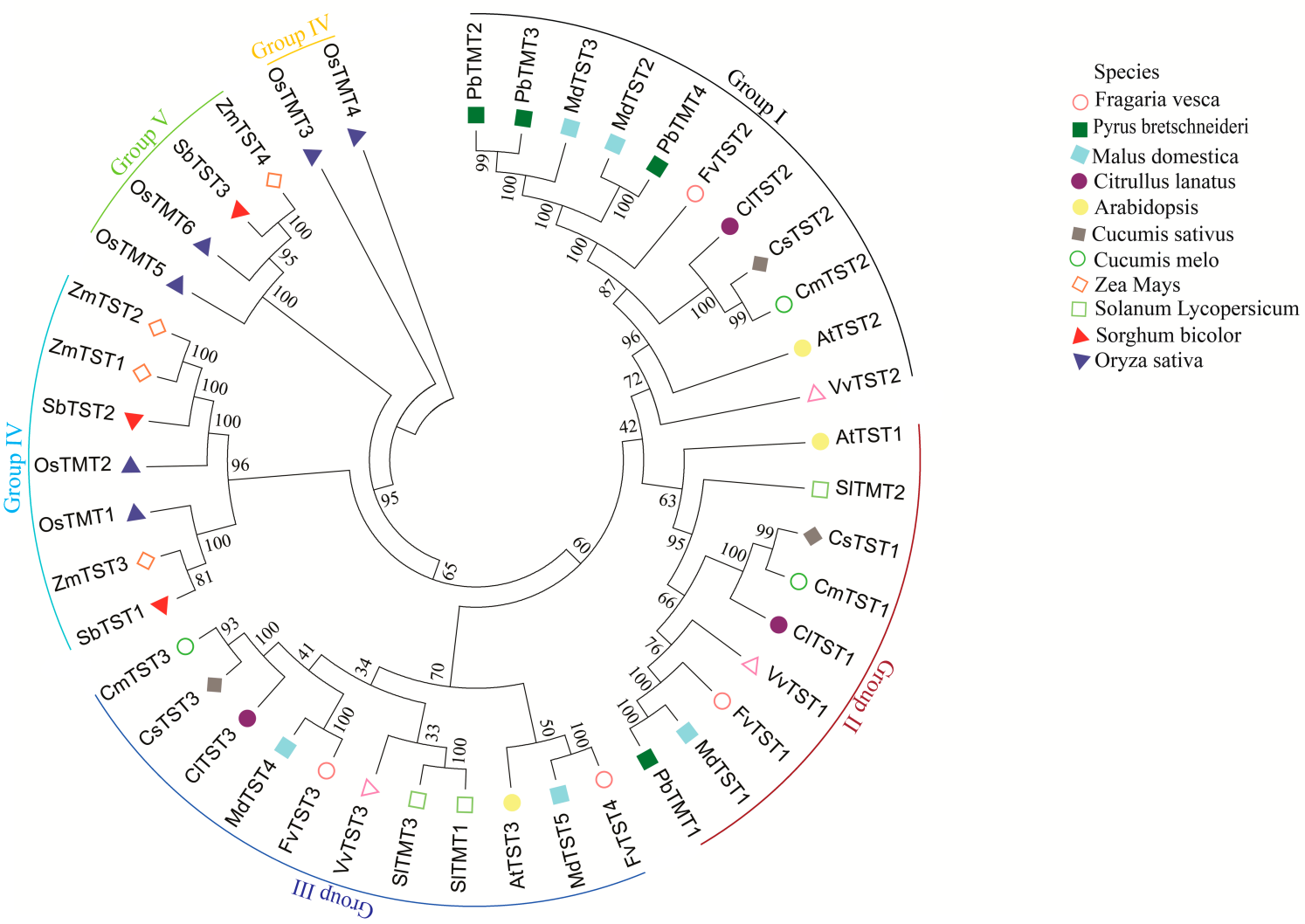


**Supplementary Figure S1.** Polygenetic analysis of the *TST*s from strawberry and other plants. Mega 5 software was used to generate the phylogenetic tree using the neighbor joining method from diverse plant species. *FvTST1*, FvH4_5g25950; *FvTST2*, FvH4_1g10110; *FvTST3*, FvH4_1g10120; *FvTST4*, FvH4_2g16850; *PbTMT1*, XP_009372620.1; *PbTMT2*, XP_018499579.1; *PbTMT3*, XP_009341490.1; *PbTMT4* XP_009340800.1, *MdTST1*, MDP0000381084; *MdTST2,* MDP0000212510; *MdTST3*, MDP0000868028; *MdTST4*, MDP0000250193 *MdTST5* MDP0000250194, *ClTST1*, Cla021919; *ClTST2*, Cla000264; *ClTST3*, Cla014359, *AtTST1*, NP_001319055.1; *AtTST2*, NP_001190923.1; *AtTST3*, NP_190717.1, *CsTST1*, Csa6G355400; *CsTST2*, Csa6G118280, *CsTST3*, Csa6G509700, *CmTST1*, MELO3C026522P1; *CmTST2* MELO3C013489P1; *CmTST3* MELO3C007980P1; *ZmTST1*, GRMZM2G126555_T01, *ZmTST2*, GRMZM2G012923_T01; *ZmTST3*, RMZM2G083173_T01; *ZmTST4*, GRMZM2G040871_T01; *SlTMT1*, Solyc03g032040.2; *SlTMT2*, Solyc04g082700.2; *SlTMT3*, Solyc02g082410.2; *OsTMT1*, XP_015625962.1; *OsTMT2*, AAG46115.1; *OsTMT3*, XP_015631484.1; *OsTMT4*, ABA94776.1 *OsTMT5*; XP_015624578.1; *OsTMT6*, XP_015616531.1.

**
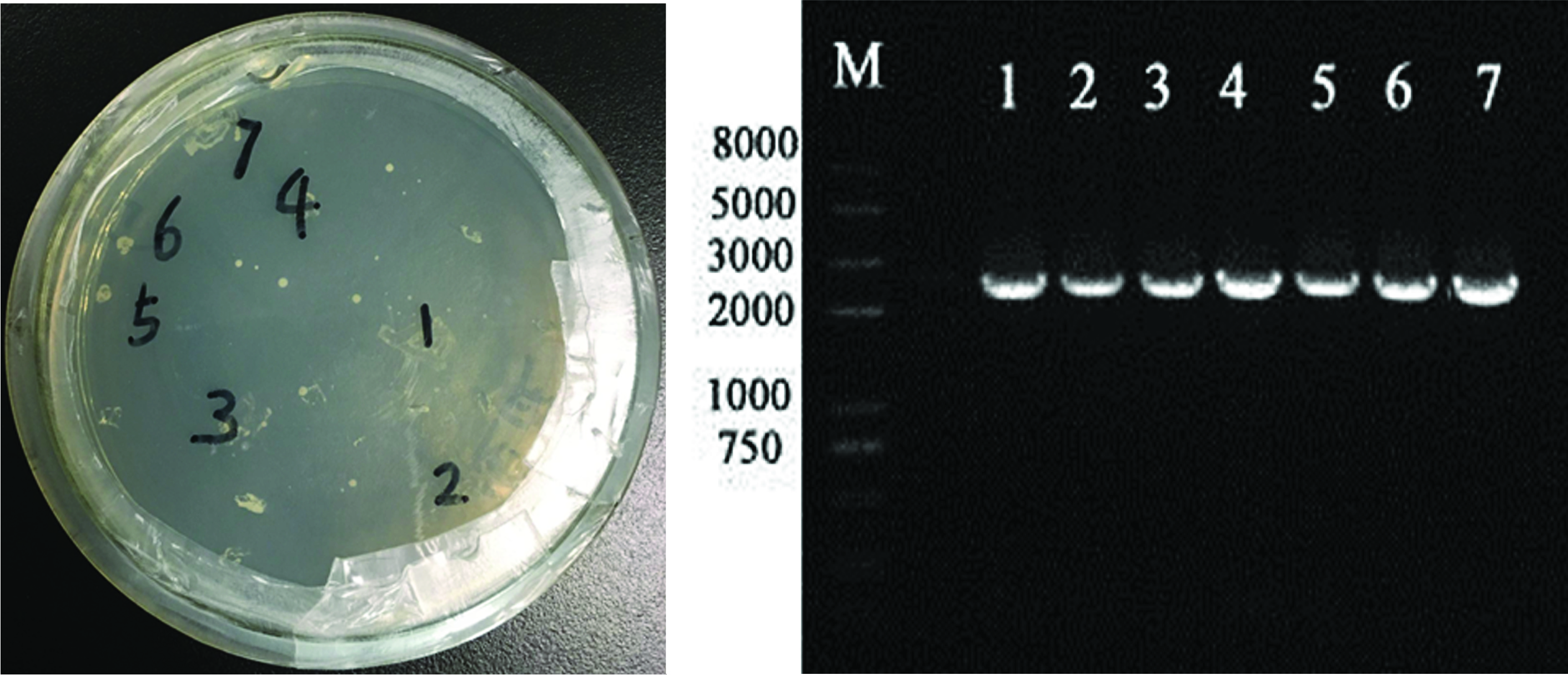
**

**Supplementary Figure S2.** PCR confirmation of *FvTST1* in hetrologous yeast system.


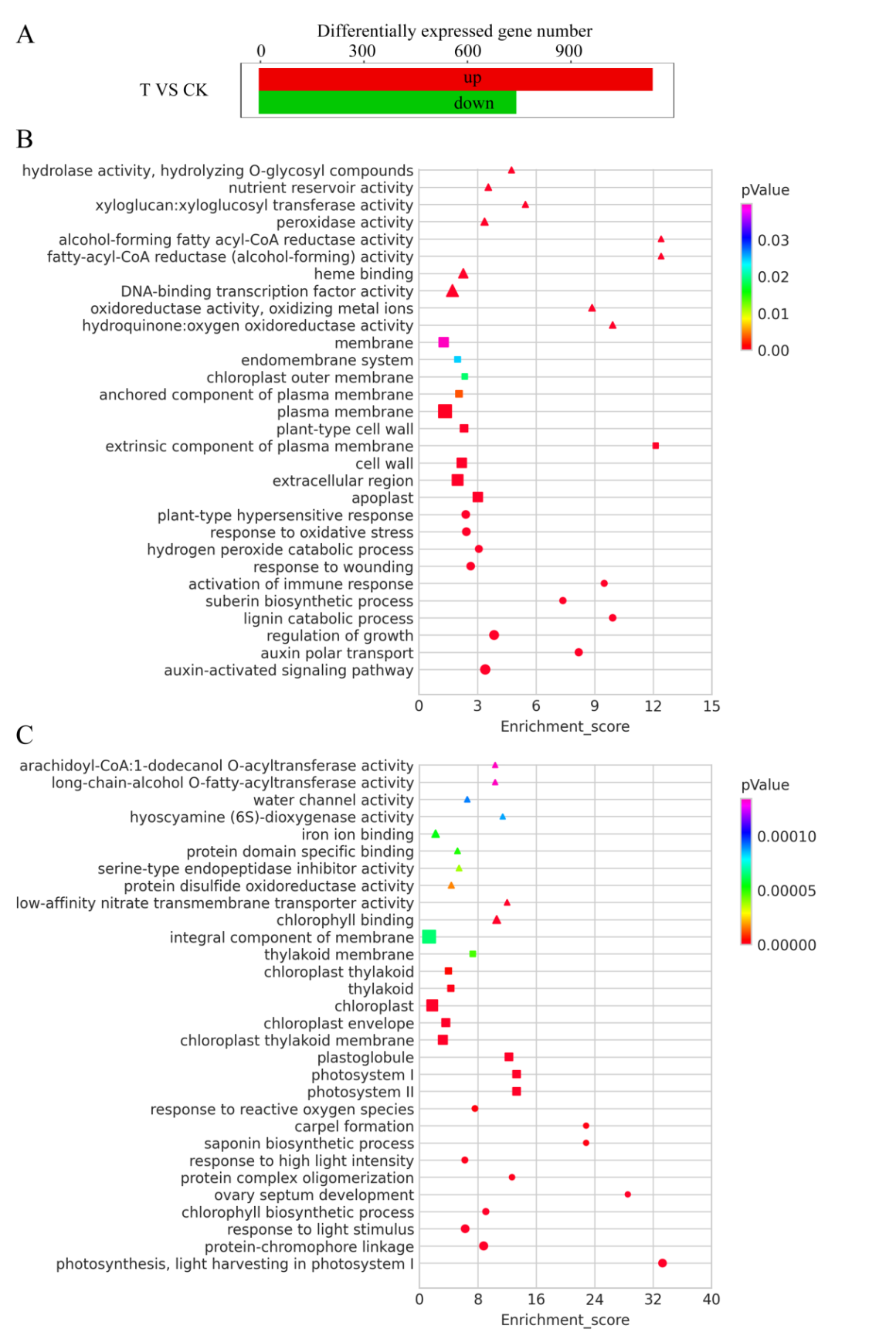


**Supplementary Figure S3.** GO enrichment analysis of differentially expressed genes. (**A**) Number of DEGs. (**B**) GO enrichment analysis of up-regulated genes. (**C**), GO enrichment analysis of down-regulated genes.


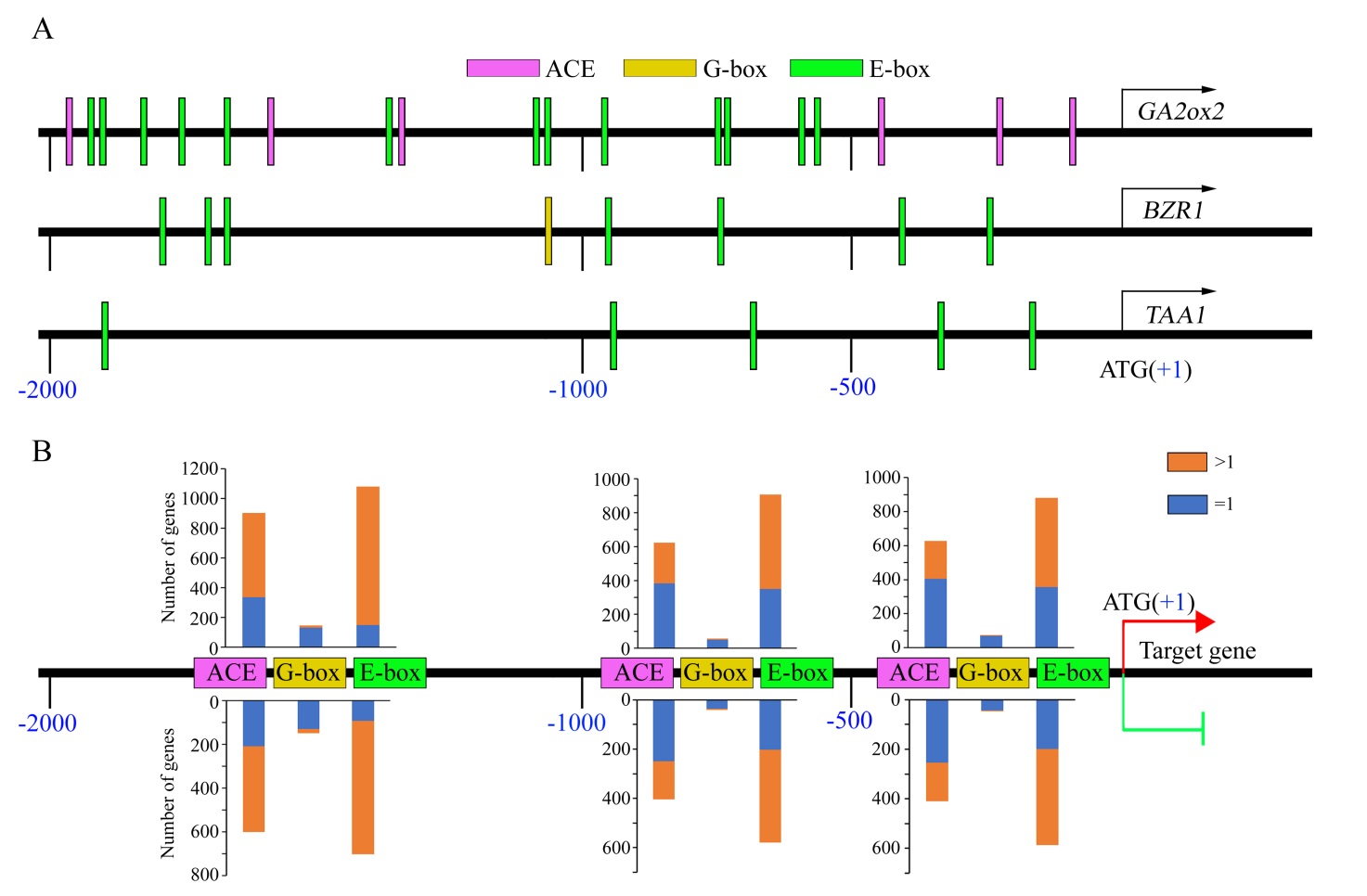


**Supplementary Figure S4**. (**A**) Graphical representation of *PIF1*, *-3*, *-4* putative binding *cis-*elements on the promoter of *GA2ox2*, *BZR1* and *TAA1*. (**B**) *PIF1*, *-3*, *-4* putative binding *cis*-elements on all up- and down-regulated genes.

**Supplementary Table S1** Quality and assembly information of the transcriptomic data

| Sample | CleanReads | ValidBases | Q30 | GC | Total reads | Uniquely mapped | Total mapped reads |
| --- | --- | --- | --- | --- | --- | --- | --- |
| CK1 | 46.82M | 93.60% | 94.97% | 43.88% | 46824386 | 94.17% | 97.91% |
| CK2 | 48.02M | 93.91% | 94.83% | 43.93% | 48016772 | 93.97% | 97.79% |
| CK3 | 48.28M | 94.21% | 94.92% | 44.07% | 48280392 | 93.84% | 97.82% |
| Set1 | 47.32M | 93.84% | 95.10% | 43.81% | 47318238 | 94.03% | 97.61% |
| Set2 | 49.29M | 92.63% | 94.65% | 43.83% | 49289284 | 94.10% | 97.85% |
| Set3 | 46.35M | 93.14% | 94.73% | 43.77% | 46353064 | 94.37% | 97.95% |

**Supplementary Table S2** Sequence information of primers

| Name | Sequence | Function |
| --- | --- | --- |
| FvTST1-F  FvTST1-R | 5’GGTACCATGAAGGGGGCTGTGTTTGTGG 3’  5’TCTAGATTACTCATTTTTGGCAGCAGCAATT3’ | Plant expression |
| FvTST1-F  FvTST1-R | 5’ GAGAACACGGGGGACTCTAGAATGAAGGGGGCTGTGTTTGTG 3’  5’ CGATCGGGGAAATTCGAGCTCTTACTCATTTTTGGCAGCAGCA 3’ | Plant expression |
| PTPRP-F  PTPRP-R | 5’GACCATGATTACGCCAAGCTTAATTAACTTGATTTTGAGTCCATGTATTT 3’  5’ ACCACCCGGGGATCCTCTAGAGGGCAATGAACAAAGTTCCAA 3’ | Promoter |
| FvTST1-F  FvTST1-R | 5’ GCGCAACAGCAGTCTCATGC 3’  5’ GGACTCCCTCCTGGTGCAAA 3’ | qPCR |
| FvTST2-F  FvTST2-R | 5’GCCACAGTGATCACAACATTTTCAG 3’  5’ACAGCTAGTCCAATCCCAAAACCAT 3’ | qPCR |
| FvTST3-F  FvTST3-R | 5’GGTAACATGTTGCAAGGATGGGATA 3’  5’GGAGACCACAACATTACCAGACCAC 3’ | qPCR |
| FvTST4-F  FvTST4-R | 5’CATGGGAGCATGGCAAGTAAAAG 3  5’CCTAGGTTGATTCCCTCCCACAC 3’’ | qPCR |
| FvTST1-F  FvTST1-R | 5’GCGGCCGAAGCTTGTAAAGAAATGAAGGGGGCTTGTTTGTGG 3’  5’ GCGGCGCTTACTCATTTTGGCAGCAGCAATT 3’ | Yeast |
| PIF1-F  PIF1-R | 5’TACAGAATTTTGGGCATTTTTCACG 3’  5’CATGGCGGTAACTGGTGTTACATTT 3’ | qPCR |
| PIF3-F  PIF3-R | 5’TGCGTCGATACCTACCCTAGAATCA 3’  5’ACCTATTTCCAGGCATGTTTTGCTT 3’ | qPCR |
| PIF4-F  PIF4-R | 5’GATGTTCCCTGGTGTCCAACACTAC 3  5’CATTGCAGCTGCTTGATTATTAGGG 3’’ | qPCR |
| BZR1-F  BZR1-R | 5’TTTTGAAGGCTCTTTGTGTTGAAGC 3’  5’AACTTGGTAAGATGGAATCGGGCTA 3’ | qPCR |
| GA2OX1-F  GA2OX1-R | 5’CTTCGTCAATGTTGGTGACTCATTG 3’  5’TTCTCCTTCCATTAGTGATGCCAAA 3’ | qPCR |
| XTH30-F  XTH30-R | 5’GGAAATGGAAGCACACATAGAGGAA 3’  5’CTCCACCCATTGCATCATTTCTTAC 3’ | qPCR |
| XTH1-F  XTH1-R | 5’TTTGTGGGTATCCTAGAAGGCCAGT 3’  5’TTCTTTGACTGAAATCCAGCTCCTG 3’ | qPCR |
| XTH23-F  XTH23-R | 5’GTCAAGGCAAAGGAAACAGAGAACA 3’  5’CATGGGTTGGTTCTTTGGATATGAA 3’ | qPCR |
| XTH23-F  XTH23-R | 5’GTCAAGGCAAAGGAAACAGAGAACA 3’  5’CATGGGTTGGTTCTTTGGATATGAA 3’ | qPCR |
| XTH27-F  XTH27-R | 5'ATGGAAATGGTAGCACAAATGTTGG 3'  5'GGAAGTCCTCACTCATTGCTTGTGT 3' | qPCR |
| XTH25-F  XTH25-R | 5'TTCCCTCGATAGAAGTTCTGGCTCT 3'  5'TGGTTCTCCACTTACATTCCCAAGA 3' | qPCR |
| XTH15-F  XTH15-R | 5'TTGTTTGGAACTCTCAACGGATCAT 3'  5'CTCGCGTAAACTCTCATTGCTTGAT 3' | qPCR |
| XTH26-F  XTH26-R | 5'TACCGGTACCAAGCATGATGAAATC 3'  5'CGTTAGGGTTCCAATGAATGGTGTA 3' | qPCR |
| EXLA1-F  EXLA1-R | 5'TCGCCGATGTTGAATACAAAAGAGT 3'  5'ATCCAACCTGAGCAACATCAACACT 3' | qPCR |
| EXLB1-F  EXLB1-R | 5'TTGTGGATTTGGTGAATATGGAAGG 3'  5'TGGTCACCTTCACCATGATCTGTTA 3' | qPCR |
| EXPA18-F  EXPA18-R | 5'AGCCACAAACCTTTGTCCACCTAAT 3'  5'CTGCGATACGAGACTGGGACAATAC 3' | qPCR |
| SAUR64-F  SAUR64-R | 5'TGTCAAGTGGTGGCCCTATTACATT 3'  5'TTCTTGGAGCAGAGAAGAAGTCGAA 3' | qPCR |
| SAUR63-F  SAUR63-R | 5'AAACTCATCAAGATGGCCAGGAAAT 3'  5'CATTTTCAAGGTAAGCCAAGGGAAC 3' | qPCR |
| WAG2-F  WAG2-R | 5'GACCCGAATTGGTCTGCTATTAAGG 3'  5'TAAGCGAATCCCGATCGATAACTTT 3' | qPCR |
| SAUR21-F  SAUR21-R | 5'AGATGTTCCCAAGGGTCACTTTGTT 3'  5'ATCCTCACTGCAGGGAATAGTCACA 3' | qPCR |
| SAUR24-F  SAUR24-R | 5'CAGAAGAAGAGATTCGTCGTGCCTA 3'  5'CTCAAGCGGTATGTGAGATCAACAA 3' | qPCR |
| SAUR19-F  SAUR19-R | 5'ACCATGGCTATCCGTATGTCTCGTA 3'  5'AAGCCAAATTCTTCCTCAGCTTGAC 3' | qPCR |
| IAA29-F  IAA29-R | 5’TCTTTGTTCATATGGAATGGCCAAC 3’  5’ACCTGCATGACCTTGATGATGAAAT 3’ | qPCR |
