# Supplementary material for: The Gene FvTST1 From Strawberry Modulates Endogenous Sugars Enhancing Plant Growth and Fruit Ripening
Source: Front Plant Sci. 2022 Jan 11;12:774582. doi: 10.3389/fpls.2021.774582 (PMC8786802; doi:10.3389/fpls.2021.774582)
Supplement: Supplementary file 2 [file Table_1.pdf]

Table S3 Gene name and IDs selected during this study and are available on https://solgenomics.net/

**Gene ID**	**Name**	**Gene ID**	**Name**
Solyc05g031600	TAA1	Solyc04g052960	SAUR67
Solyc06g008030	PIF1	Solyc04g052990	SAUR67
Solyc07g043580	PIL13	Solyc04g053020	SAUR67
Solyc01g102300	PIL15	Solyc04g053030	SAUR67
Solyc05g053410	PHYB	Solyc10g052550	SAUR67
Solyc04g079980	BZR1	Solyc10g052560	SAUR67
Solyc01g081060	XTH30	Solyc10g052590	SAUR67
Solyc01g099630	XTH1	Solyc11g011630	SAUR67
Solyc03g093080	XTH23	Solyc11g011640	SAUR67
Solyc03g093130	XTH23	Solyc11g011660	SAUR67
Solyc04g008210	XTH27	Solyc11g011710	SAUR67
Solyc07g009380	XTH25	Solyc11g011730	SAUR67
Solyc07g056000	XTH15	Solyc04g052980	SAUR64
Solyc12g007250	XTH26	Solyc10g052530	SAUR64
Solyc01g112000	EXLA1	Solyc04g053010	SAUR63
Solyc08g077900	EXLB1	Solyc07g056400	WAG2
Solyc08g080060	EXPA18	Solyc01g110710	SAUR21
Solyc07g056670	GA2OX2	Solyc01g110903	SAUR21
Solyc04g052980	SAUR64	Solyc01g110930	SAUR21
Solyc04g053010	SAUR63	Solyc04g052970	SAUR21
Solyc04g053030	SAUR67	Solyc11g011650	SAUR21
Solyc07g056400	WAG2	Solyc11g011720	SAUR21
Solyc01g110903	SAUR21	Solyc01g110720	SAUR21
Solyc04g053000	SAUR24	Solyc01g110880	SAUR20
Solyc01g110790	SAUR19	Solyc10g052540	SAUR24
Solyc08g021820	IAA29	Solyc04g053000	SAUR24
Solyc02g081330	PSY1	Solyc10g150147	SAUR32
Solyc03g033510	CSI3	Solyc01g110570	SAUR50
Solyc03g096545	PLAT1	Solyc01g110790	SAUR19
Solyc04g005660	PRE6	Solyc01g110793	SAUR19
Solyc04g056310	MYB93	Solyc01g098400	AIP15a
Solyc04g074170	MYB93	Solyc02g036370	RVE1
Solyc05g051550	MYB93	Solyc02g063450	GRDP2
Solyc01g096860	WRI3	Solyc04g054280	IAA4
Solyc09g092560	CYP83B1	Solyc07g008020	IAA20
Solyc09g092580	CYP83B1	Solyc12g005310	GH3.6
Solyc09g092600	CYP83B1	Solyc07g066330	NAC021
Solyc10g079840	PRFB3	Solyc10g081180	BG3
Solyc08g006460	HIP1	Solyc07g053030	GH3.6
Solyc06g061160	ZF10	Solyc03g098320	RVE1
Solyc04g052890	SAUR67	Solyc12g095750	PILS1
Solyc12g097040	SMAP1	Solyc03g123410	ABP19A
